# A novel cuproptosis-related immune checkpoint gene signature identification and experimental validation in hepatocellular carcinoma

**DOI:** 10.1038/s41598-022-22962-y

**Published:** 2022-11-02

**Authors:** Yusai Xie, Wei Zhang, Jia Sun, Lingyan Sun, Fanjie Meng, Huiying Yu

**Affiliations:** 1 Laboratory of Basic Medicine, General Hospital of Northern Theatre Command, Shenyang, 110016 Liaoning China; 2 Department of Hepatobiliary Surgery, General Hospital of Northern Theatre Command, Shenyang, 110016 Liaoning China

**Keywords:** Cancer genetics, Cancer therapy, Immune evasion, Immunotherapy, Prognostic markers, Tumour biomarkers, Gene regulation, Bioinformatics

## Abstract

Copper-induced death, also termed cuproptosis, is a novel form of programmed cell death and is promising as a new strategy for cancer therapeutics. Elevated copper levels in tumor cells are positively associated with high PD-L1 expression. Nonetheless, the prognostic significance of cuproptosis-related immune checkpoint genes (CRICGs) in hepatocellular carcinoma remains to be further clarified. This study aimed to construct the prognostic CRICG signature to predict the immunotherapy response and outcomes of HCC patients. The co-expressed CRICGs were first screened through Pearson correlation analysis. Based on the least absolute shrinkage and selection operator-COX regression analyses, we identified a prognostic 5-CRICGs model, which closely correlates with poor outcomes, cancer development, and immune response to hepatocellular carcinoma. External validation was conducted using the GSE14520 dataset. Lastly, qRT-PCR was performed to determine the expression of the CRICGs in HCC. In summary, we developed and validated a novel prognostic CRICG model based on 5 CRICGs. This prognostic signature could effectively forecast the outcomes and immune response of HCC patients, which may serve as biomarkers for anticancer therapy.

## Introduction

Hepatocellular carcinoma (HCC) is a common malignant tumor with a high mortality rate globally. Even though early screening and multidisciplinary diagnosis of HCC have greatly improved, the survival rate of postoperative HCC patients remains unsatisfactory^1^. Recently, immune checkpoint blockade (ICB) therapy, specifically targeting PD-1/PD-L1, has brought revolutionary progress in the advanced HCC treatment^2^. Still, only a minority of HCC patients benefit from current ICB therapies. The overall response rate (ORR) of ICB monotherapy is approximately 20%, while the ORR after combination treatment increase to almost 30%^3^. Immunotherapy based on the application of immune checkpoint inhibitors (ICIs), as single agents or combined with chemotherapeutic drugs, anti-angiogenic agents, and kinase inhibitors, offers great promise for HCC treatment^4^. Therefore, it is of great significance to focus on the expression pattern of immune checkpoint genes (ICGs) and develop more relevant predictive biomarkers to predict the therapeutic response and prognosis of HCC.

Copper (Cu) is an important metallic trace element in the human body and is engaged in various biochemical functions. Disorder of Cu homeostasis is associated with diverse pathologies, like Menkes disease, hepatic Wilson disease^5^, and even tumorigenesis^6^. Several studies have found that Cu levels are significantly upregulated in tumor tissue and serum of cancer patients^7–13^. Moreover, alteration of intracellular Cu levels may be involved in cancer development^14^. Recently, a novel mechanism of Cu-dependent cell death, also termed cuproptosis, was reported by Tsvetkov et al.^15–17^. It was proposed that cuproptosis is attributed to intracellular Cu accumulation inducing aggregation of lipoylated tricarboxylic acid (TCA) cycle components and resulting in proteotoxic stress and cell death^16^.

Cu homeostasis is essential for maintaining normal immune function^18–20^. Recent studies found that elevated Cu levels in tumor cells contribute to immune escape by enhancing PD-L1 expression^21^. The relationship between cuproptosis and ICGs has not been elucidated. Therefore, studying the prognostic signature of cuproptosis-related immune checkpoint genes (CRICGs) is of great interest. Based on The Cancer Genome Atlas (TCGA) and Gene expression omnibus (GEO) databases, we identified and validated the prognostic CRICG model. In the meantime, we evaluated the potential correlation between this signature and clinicopathological characteristics, tumor immunity, and somatic mutation.

## Methods

### Data acquisition and identification of cuproptosis-related ICGs

The transcriptome RNA‐seq dataset (HT Seq-TPM) and the corresponding clinical information of 424 HCC samples (including 374 tumor samples and 50 normal samples) were available from TCGA database (https://portal.gdc.cancer.gov). The progression-free survival (PFS) information of TCGA-HCC samples was acquired from the UCSC Xena platform (https://xenabrowser.net/). According to the research of Tsvetkov et al.^16^, we focused on 13 cuproptosis-related genes (CRGs). These 13 CRGs included 7 cuproptosis resistant genes (DLD, DLAT, FDX1, LIAS, LIPT1, PDHA1, and PDHB), as well as 3 other key enzymes in the TCA cycle (DBT, GCSH, and DLST) and 3 Cu transport-related factors (SLC31A1, ATP7A and ATP7B). The profile of 79 immune checkpoint genes was obtained as previously reported^22^. R package *limma* was applied to extract the gene expression profile of 13 CRGs and 79 ICGs from TCGA database. The CRICGs co-expressed with 13 CRGs were determined with the criteria of Pearson correlation coefficient > 0.2 and *p*-value < 0.001. The predictive CRGs-CRICGs co-expression network was established using Cytoscape software (https://cytoscape.org).

### Research strategy

A flowchart of our study was carried out (Fig. S1). In brief, the gene expression profile of 13 CRGs and 79 ICGs was extracted from the TCGA database. Then, the predictive CRICGs were determined according to Pearson correlation analysis. Subsequently, the overall HCC cohort (n = 370) was randomly classified into the training (n = 185) and testing (n = 185) cohorts. LASSO-COX analysis was performed on the training group to construct the prognostic risk model. Subsequently, internal prognostic validation was performed for the testing and the overall cohorts. Furthermore, clinical correlation, pathway prediction, immune landscape, and TMB correlation analyses were applied to study the potential biological functions of this CRICG signature. Finally, external validation and experimental validation were adopted to verify this signature.

### Establishment of CRICG signature

Based on the relevant clinical information, the overall HCC cohort (n = 370) was randomly classified into training and testing cohorts with a ratio of 1:1. The Chi-square test were utilized to verify the effectiveness of random assignment. First, univariate Cox regression analysis was performed on the training cohort for filtering prognostic CRICGs. Next, LASSO regression analysis was adopted to screen CRICGs with the minimum ten-fold cross‐validation using R package *glmnet*^23^. Further, multivariate Cox regression analysis was employed to screen CRICGs and determine the corresponding risk regression coefficients. Thus, a prognostic risk model based on 5-ICGs was established. The following equation was utilized to compute the risk score:

$${\text{Risk}}\,\,{\text{score }} = \mathop \sum \limits_{i = 1}^{n} coefi \times expi$$

The *expi* and *coefi* indicate the expression levels of ICG and its corresponding coefficients, respectively.

### Internal validation of CRICGs signature

The training, testing, and overall cohort of HCC patients were separated into high- and low-risk populations based on the median value of the training risk score. Kaplan–Meier (K–M) survival analyses were carried out using R package *survival* to evaluate overall survival (OS) values. K–M survival analyses were also performed on subgroups stratified by different clinicopathological characteristics. The independent variables of the risk model were evaluated sequentially by Cox regression analysis. Subsequently, the ROC curves were utilized to assess the validity of this prognostic model by R package *timeROC*^24^. The concordance index (C-index) was applied to evaluate the predictive capability of this prognostic model.

### Nomogram establishment

Based on the risk group and clinicopathological characteristics, the nomogram of 1-, 3- and 5-years OS was established via R package *rms*. In addition, the corresponding calibration curves were applied to illustrate the consistency level of the prediction results.

### Clinical correlation of prognostic CRICGs signature

The Chi‐square test and Wilcox signed-rank test were applied to study the correlation between risk groups and clinicopathologic factors. The heatmap displayed the clinical correlation findings of the Chi-square test. The boxplot depicted the clinical correlation data of the Wilcox signed-rank test.

### Functional enrichment analyses

The three-dimensional (3D) distribution patterns of risk sample classification in different models were presented by principal component analysis (PCA). With the criteria of |log fold change|> 1 and false discovery rate (FDR) < 0.05, the differentially expressed genes (DEGs) among the low- and high-risk populations were determined through R package *limma*. In order to study the relevant biological processes engaged in this prognostic signature, Gene Ontology (GO) and Kyoto Encyclopedia of Genes and Genomes (KEGG)^25^ analyses were performed on the identified DEGs using R package *clusterProfiler* (*p* < 0.05). Furtherly, Gene Set Enrichment Analysis (GSEA) was used to determine the biological functions and pathways significantly enriched in high‐ and low‐risk populations (FDR < 0.05).

### Assessment of immune cell infiltration

The tumor microenvironment (TME) scores of HCC samples were evaluated by R package *ESTIMATE*^26,27^. TME scores, including estimate scores, immune scores, and stromal scores, could infer tumor purity in tumor tissue. The immune infiltration scores of TCGA samples quantified by multiple algorithms (MCP-counter, quanTIseq, EPIC, CIBERSORT-abs, CIBERSORT, xCell, and TIMER) were obtained from the TIME platform (http://timer.cistrome.org)^28^. Using the single-sample gene set enrichment analysis (ssGSEA) method with R package *GSVA*, the degree of 16 immune cells infiltration and the activity level of 13 immune-related functions were evaluated in HCC samples^29^. The differential expression between risk groups and ICG expression was analyzed via R package *limma.* Tumor Immune Dysfunction and Exclusion (TIDE, http://tide.dfci.harvard.edu/) platform was applied to forecast the biomarker sensitivity to ICB therapies in tumors like melanoma and non-small cell lung cancer^30^. The TCGA-HCC expression profiles were imported into the TIDE platform to obtain the TIDE scores of HCC samples. The poor ICB response is associated with high TIDE and dysfunction prediction scores in tumors, as well as low exclusion scores.

### Drug susceptibility prediction

To further assess the significance of the prognostic signature in predicting clinical HCC therapy response, the half-maximal inhibitory concentration (IC50) values for chemotherapeutic or targeted drugs in high- and low-risk populations were evaluated via the R package *pRRophetic*. The Wilcoxon signed-rank test was applied to compare the differences in IC50 values between low- and high-risk populations (*p* < 0.01).

### Somatic mutation analysis and TMB correlation analysis

The simple nucleotide variation information of HCC samples was acquired from the TCGA database. Then, the gene mutation type, frequency, and tumor mutation burden (TMB) scores of HCC samples were calculated using Perl software. The mutation genes among high- and low-risk populations were identified and visualized by R package *maftools*^31^.

### External validation of CRICGs signature

GSE14520 dataset based on GPL3921 platform was available from the GEO database (https://www.ncbi.nlm.nih.gov/geo/). The GSE14520 dataset, containing 221 HCC samples with microarray gene expression and survival information, was adopted to validate the prognostic significance of this CRICG signature externally. K–M survival analyses of OS and PFS were conducted with the optimal cut-off value. The ROC curves of 1-year OS were carried out in the GSE14520 dataset.

### Cell culture and qRT-PCR assay

The human normal liver cell (MIHA) and HCC cell (SMMC-7721) were gifts from the College of Basic Medical Sciences, China Medical University, Shenyang, China. Human HCC cell line HEPG2 was maintained in our laboratory. These cell lines were cultured in high-glucose Dulbecco's Modified Eagle Medium (DMEM, Shanghai Basal Media Technologies, China) containing 10% fetal bovine serum (FBS, Biological Industrie, Israel) at 37 °C in a humidified incubator with 5% CO_2_. As instructed by the corresponding manufacturer, RNAiso Plus (Code No. 9108, Takara) was utilized to extract the total RNA from cell lines, and the PrimeScript RT reagent kits (RR047A, Takara) were used for cDNA synthesis. TB green Master reagents (RR820A, Takara) were used for amplificated through Applied Biosystems 7500 Real-Time PCR System. The relative gene expression level was computed through a 2^−ΔΔCt^ method. All primers were synthesized by Shanghai Invitrogen Biotechnology (Shanghai, China). The primer sequences utilized are displayed in Table 1.

**Table 1:** The Primer sequences list for qRT-PCR.

Gene	Forward sequence	Reverse sequence
TNFRSF14	CCAAGTGCAGTCCAGGTTAT	ATTGAGGTGGGCAATGTAGG
TNFSF9	GAGCTTTCGCCCGACGAT	CCTCTTTGTAGCTCAGGCCC
TNFSF4	ATGAACCAACCCCTGGAAGC	GTTTGGAGGCTGGGAAAGC
TNFRSF4	GCAATAGCTCGGACGCAATCT	GAGGGTCCCTGTGAGGTTCT
CD226	GATGTTGGCTACTATTCCTGCTC	CTGAACCACCTGTATCACCTTC
ACTB	GGCACCCAGCACAATGAA	TAGAAGCATTTGCGGTGG

### Statistics analysis

The Student's t-test was used to compare the gene expression differences of qRT-PCR. All statistical analyses were carried out using R software (version 4.1.0). Unless indicated otherwise, *p*-value < 0.05 was deemed to demonstrate statistically significant.

## Results

### Identification of CRICGs

**Figure 1:**
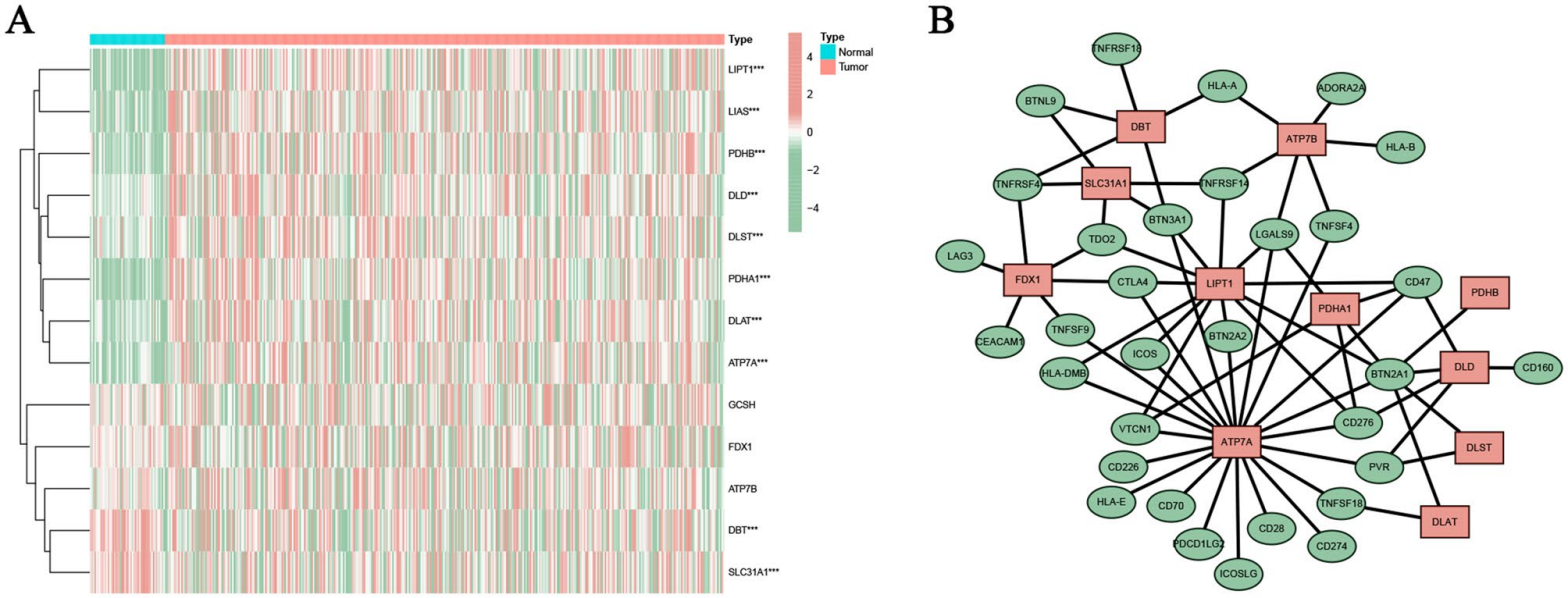
Identification of cuproptosis-related ICGs in HCC. (**A**) The expression heatmap of 13 cuproptosis‐related genes. (**B**) The co-expression network between cuproptosis-related genes and ICGs.

The expression heatmap of 13 interested CRGs was shown in Fig. 1A. Based on the Pearson correlation analysis, 32 CRICGs co-expressed with these CRGs were obtained. The predictive CRGs-CRICGs co-expression network was constructed as shown in Fig. 1B.

### Establishment and internal validation of prognostic CRICG model

**Figure 2:**
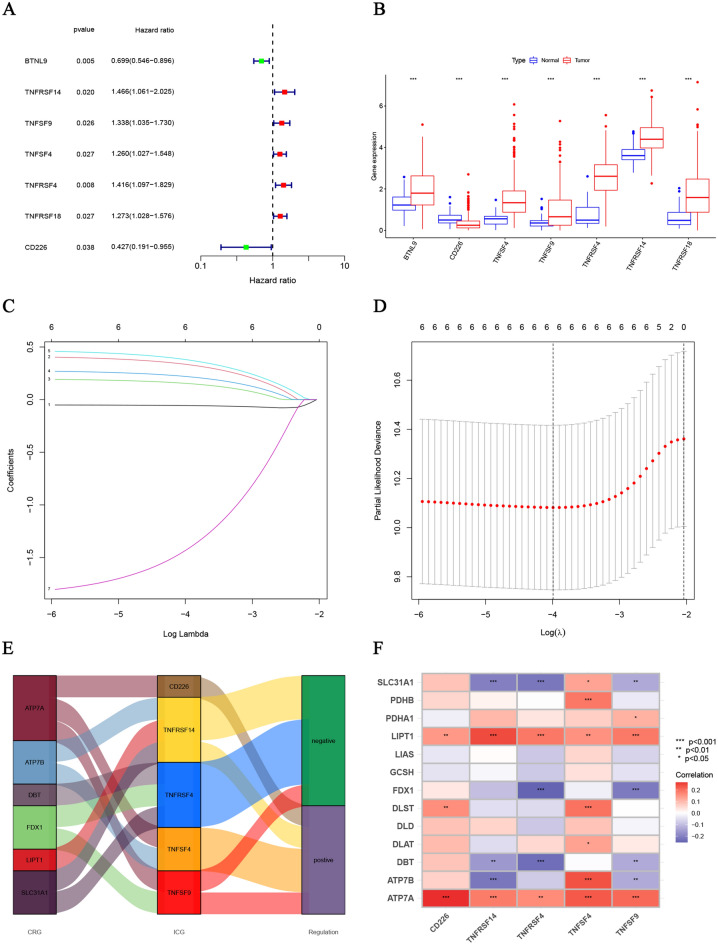
Construction of cuproptosis‐related ICGs. (**A**, **B**) The expression profiles of 7 ICGs preliminarily screened by univariate Cox regression analysis. (**C**, **D**) The cvfit and lambda curves performed by the LASSO regression model. (**E**, **F**) The Sankey diagram and the correlation heatmap of prognostic cuproptosis‐related ICGs screened by the multivariate Cox regression analysis.

The overall cohort of 370 HCC patients was randomly classified into the training and testing cohorts with a ratio of 1:1. Further, the Chi-square test verified the effectiveness of random assignment (Table 2). The training cohort was then applied to develop the prognostic model. In the first step, 7 ICGs were screened from 32 CRICGs through the univariate Cox regression analysis (Fig. 2A,B). Subsequently, 6 CRICGs were identified by LASSO regression analysis (Fig. 2C,D), 5 of which (TNFRSF4, TNFRSF14, TNFSF4, TNFSF9, and CD226) were screened by means of the multivariate Cox regression analysis (Fig. 2E,F). Then, the risk score was computed using the following equation:

$$ \begin{aligned} Risk\,\,score = & \,TNFRSF{4} \times \left( {0.{4827}} \right) \\ & + \,TNFRSF{14} \times \left( {0.{4436}} \right) \\ & + \,TNFSF{4} \times \left( {0.{28}00} \right) \\ & + \,TNFSF{9} \times \left( {0.{2}0{89}} \right) \\ & + \,CD{226} \times \left( { - {1}.{9}0{82}} \right) \\ \end{aligned} $$

**Table 2:** The detailed clinicopathologic factors information between the training and testing groups.

Covariates	Type	Total	Testing	Training	*p*-value
Age	≤ 65	232 (62.7%)	116 (62.7%)	116 (62.7%)	1
	> 65	138 (37.3%)	69 (37.3%)	69 (37.3%)	
Gender	Female	121 (32.7%)	56 (30.27%)	65 (35.14%)	0.3753
	Male	249 (67.3%)	129 (69.73%)	120 (64.86%)	
Grade	G1	55 (14.86%)	25 (13.51%)	30 (16.22%)	0.4544
	G2	177 (47.84%)	84 (45.41%)	93 (50.27%)	
	G3	121 (32.7%)	67 (36.22%)	54 (29.19%)	
	G4	12 (3.24%)	5 (2.7%)	7 (3.78%)	
	Unknown	5 (1.35%)	4 (2.16%)	1 (0.54%)	
Stage	Stage I	171 (46.22%)	88 (47.57%)	83 (44.86%)	0.5787
	Stage II	85 (22.97%)	42 (22.7%)	43 (23.24%)	
	Stage III	85 (22.97%)	42 (22.7%)	43 (23.24%)	
	Stage IV	5 (1.35%)	1 (0.54%)	4 (2.16%)	
	Unknown	24 (6.49%)	12 (6.49%)	12 (6.49%)	
T	T1	181 (48.92%)	91 (49.19%)	90 (48.65%)	0.8085
	T2	93 (25.14%)	45 (24.32%)	48 (25.95%)	
	T3	80 (21.62%)	38 (20.54%)	42 (22.7%)	
	T4	13 (3.51%)	8 (4.32%)	5 (2.7%)	
	Unknown	3 (0.81%)	3 (1.62%)	0 (0%)	
M	M0	266 (71.89%)	136 (73.51%)	130 (70.27%)	0.5936
	M1	4 (1.08%)	1 (0.54%)	3 (1.62%)	
	Unknown	100 (27.03%)	48 (25.95%)	52 (28.11%)	
N	N0	252 (68.11%)	132 (71.35%)	120 (64.86%)	0.1151
	N1	4 (1.08%)	0 (0%)	4 (2.16%)	
	Unknown	114 (30.81%)	53 (28.65%)	61 (32.97%)

**Figure 3:**
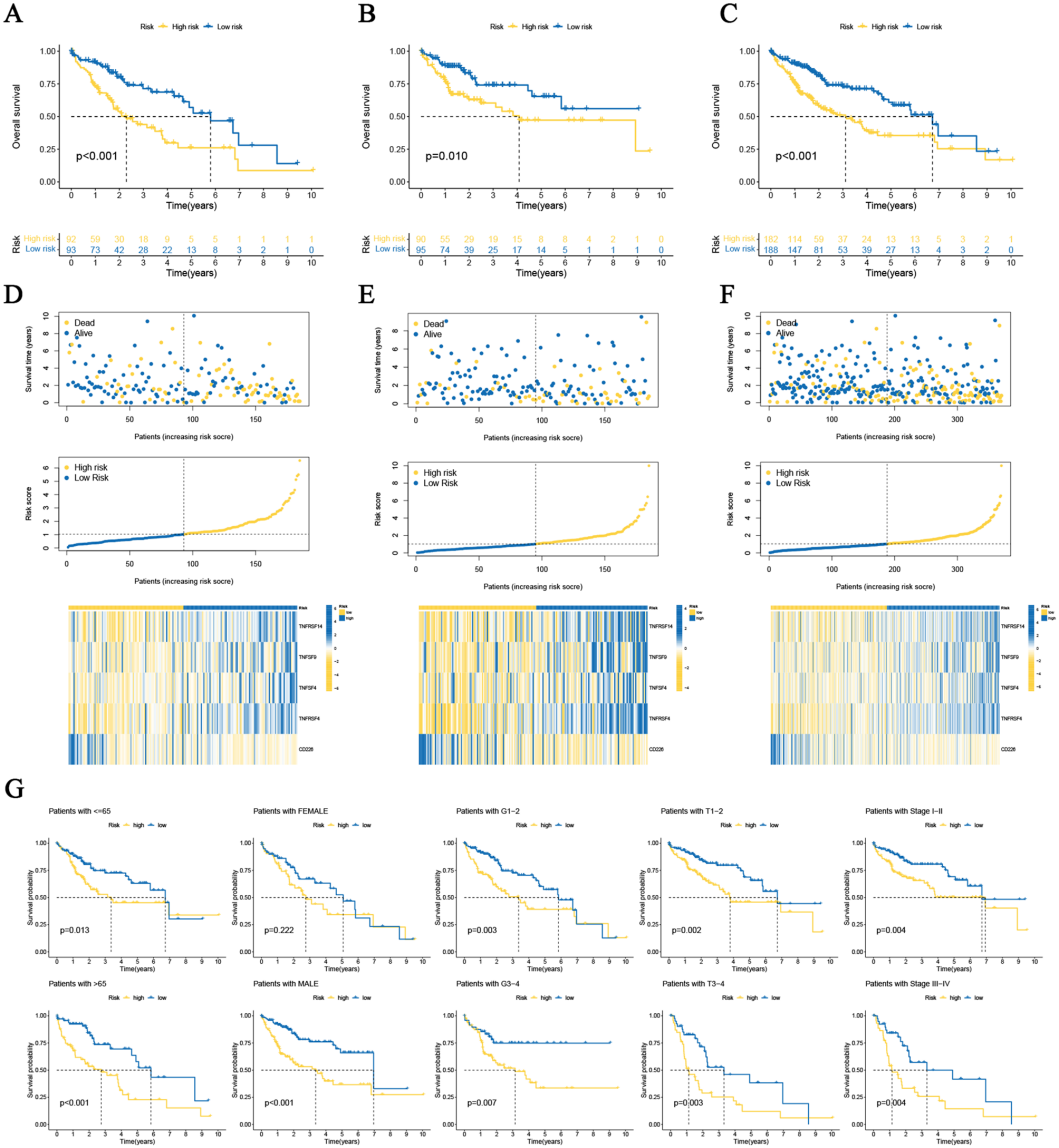
Prognosis value evaluation of cuproptosis‐related ICGs model. (**A–C**) The overall survival value of the training, testing, and overall cohorts. (**D**–**F**) The distributions of survival status, risk scores, and expression heatmap in the training, testing, and overall cohorts. (**G**) Overall survival value stratified by clinicopathologic features.

**Figure 4:**
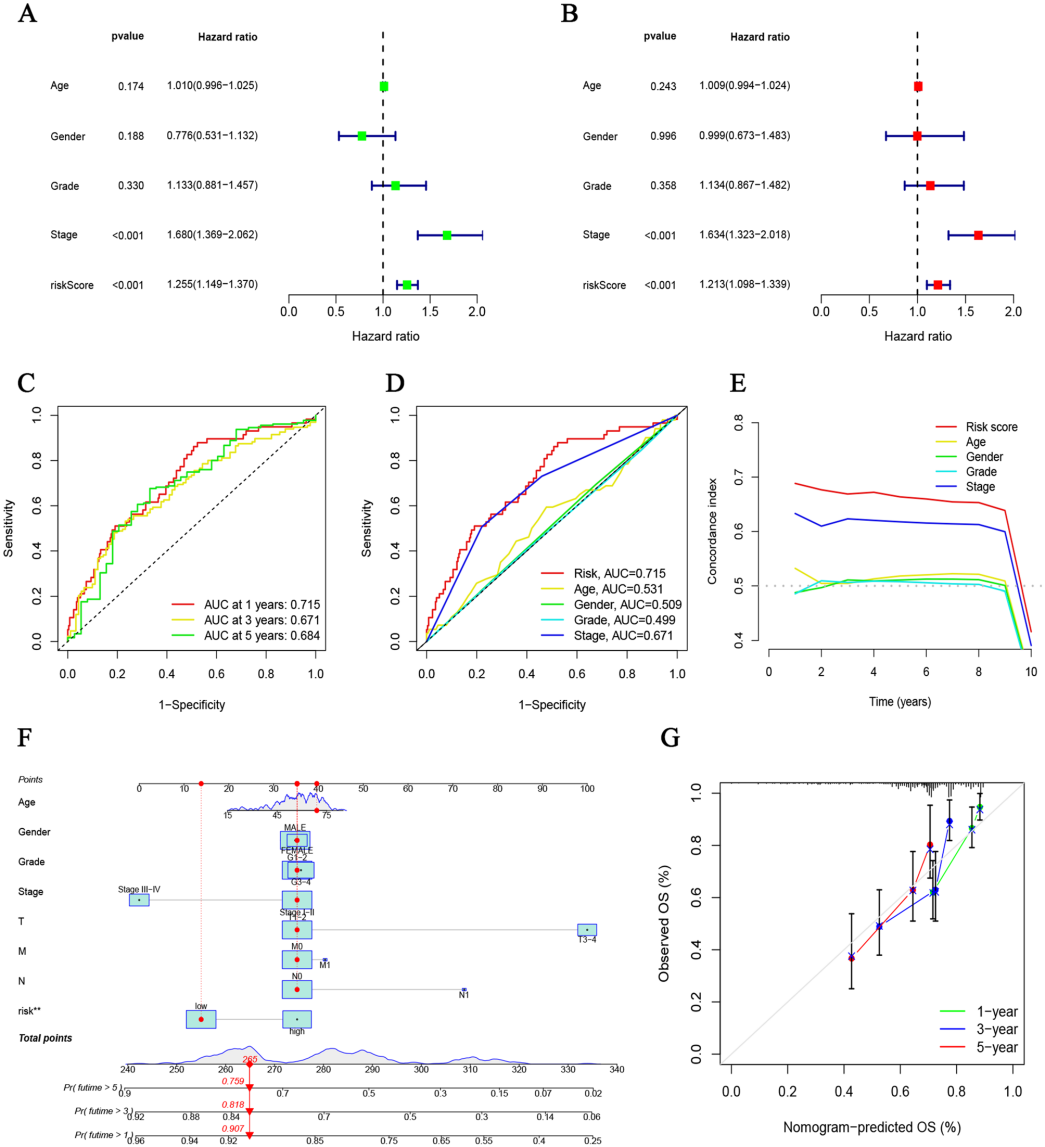
Internal validation of prognosis risk model and Nomogram demonstration. (**A**, **B**) Cox regression analyses of clinicopathologic features and risk scores. (**C**) The ROC curves for 1-, 3- and 5-years OS. (**D**, **E**) The ROC curves and C-index of risk score and clinicopathologic features. (**F**, **G**) Nomogram and calibration curves of the prognosis model.

Additionally, K–M survival analyses were applied to internally validate the prognostic significance of this 5-CRICGs model. In the training, testing, and overall HCC cohorts, the median OS periods of the high-risk populations were remarkably shorter than those of the low-risk populations (Fig. 3A–F). Similar results were observed for PFS (*p* = 0.008, Fig. S2A). Moreover, subgroups stratified by different clinicopathological characteristics suggest that the high-risk populations are strongly related to unfavorable outcomes (Fig. 3G). The hazard ratio (HR) of this risk score in multivariate Cox regression analysis was 1.213 (*p* < 0.001, Fig. 4A,B), revealing that this signature could independently predict the HCC outcomes. The ROC curves analyses of 1‐year (AUC = 0.715), 3 years (AUC = 0.671), and 5 years (AUC = 0.684) OS show the high accuracy of this prognostic signature (Fig. 4C). The ROC curves and C-index analyses of this signature were considerably superior to other clinicopathological features, indicating the strong predictive ability of this risk model signature (Fig. 4D,E).

### Construction of nomogram

Thus, we constructed a nomogram to forecast 1-, 3- and 5-years OS incidences for HCC patients (Fig. 4F). The calibration curves for each year closely correspond to the diagonal line (Fig. 4G), indicating the reliability of this prognostic model for predicting HCC patient survival.

### Clinical value of prognostic signature

**Figure 5:**
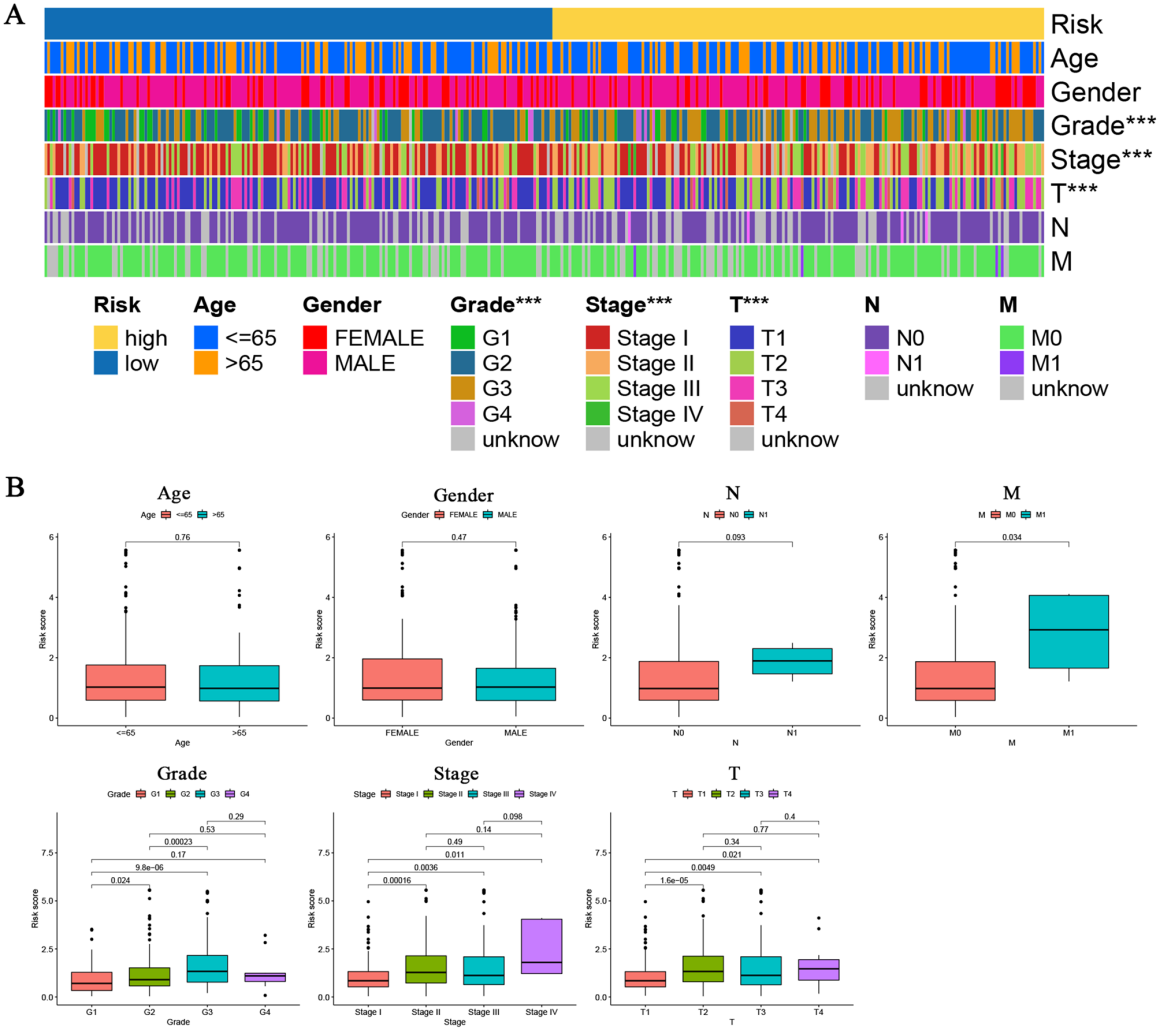
Clinical correlation analysis between the prognostic signature and clinicopathologic features by (**A**) the Chi-square test and (**B**) Wilcox signed-rank test. ***p < 0.001.

Then, we applied the Chi-square test and Wilcox signed-rank test to investigate the correlation among this prognostic signature and clinicopathological features. As illustrated in the heatmap and boxplot, this model signature was markedly related to the grade, Tumor-node-metastasis (TNM) stage, and T stage of HCC (*p* < 0.001, Fig. 5A,B). In addition, this signature was dramatically associated with the M stage of HCC through Wilcox signed-rank test (*p* = 0.034, Fig. 5B).

### Functional enrichment analyses

**Figure 6:**
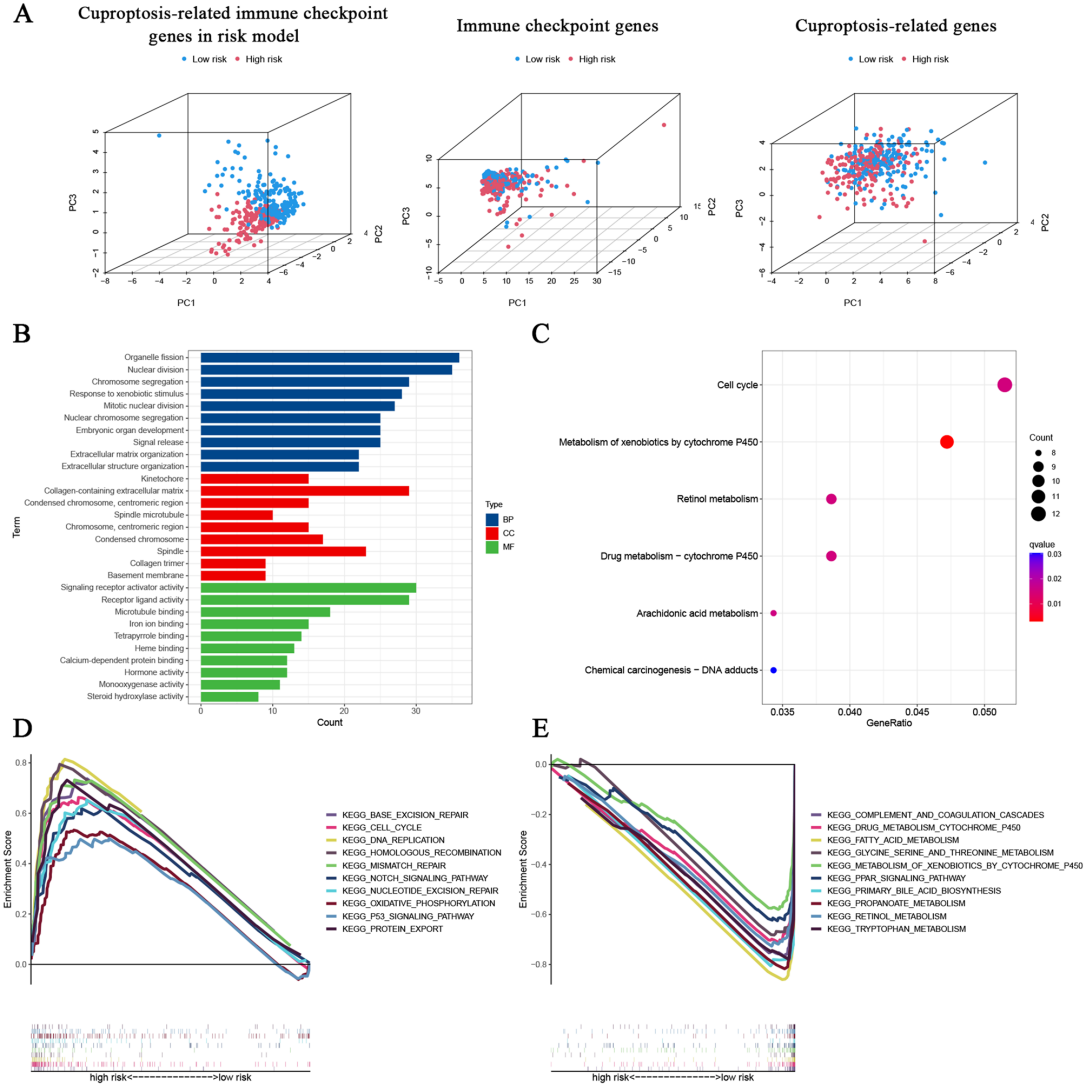
Functional analyses of cuproptosis‐related ICGs prognostic signature. (**A**) The PCA scatterplot of sample 3D distribution. (**B**) GO and (**C**) KEGG enrichment analysis of risk differential genes. (**D**, **E**) GSEA analysis of prognostic signature.

The 3D PCA analysis demonstrated the feasibility and availability of the CRICGs model in risk sample classification (Fig. 6A). Next, the DEGs between the low‐ and high‐risk populations were identified by R package *limma*. The GO analysis illustrated that these DEGs were enriched in various biological processes and molecular functions associated with cell replication, including chromosome segregation, nuclear division, and microtubule binding (Fig. 6B). The KEGG analysis suggests that these DEGs are enriched in cell cycle, drug metabolism-cytochrome p450, metabolism of xenobiotics by cytochrome p450, and retinol metabolism (Fig. 6C). The GSEA analysis indicated that high-risk populations were involved in several tumor-related pathways, such as cell cycle, DNA replication, p53 signaling pathway, and DNA repair pathways^32^(base excision repair, nucleotide excision repair, mismatch repair, and homologous recombination) (Fig. 6D). While the low-risk populations were involved in many kinds of immune or metabolic pathways, including complement and coagulation cascades, retinol metabolism, fatty acid metabolism, and PPAR signaling pathway (Fig. 6E).

### Immune-related analysis of prognostic signature

**Figure 7:**
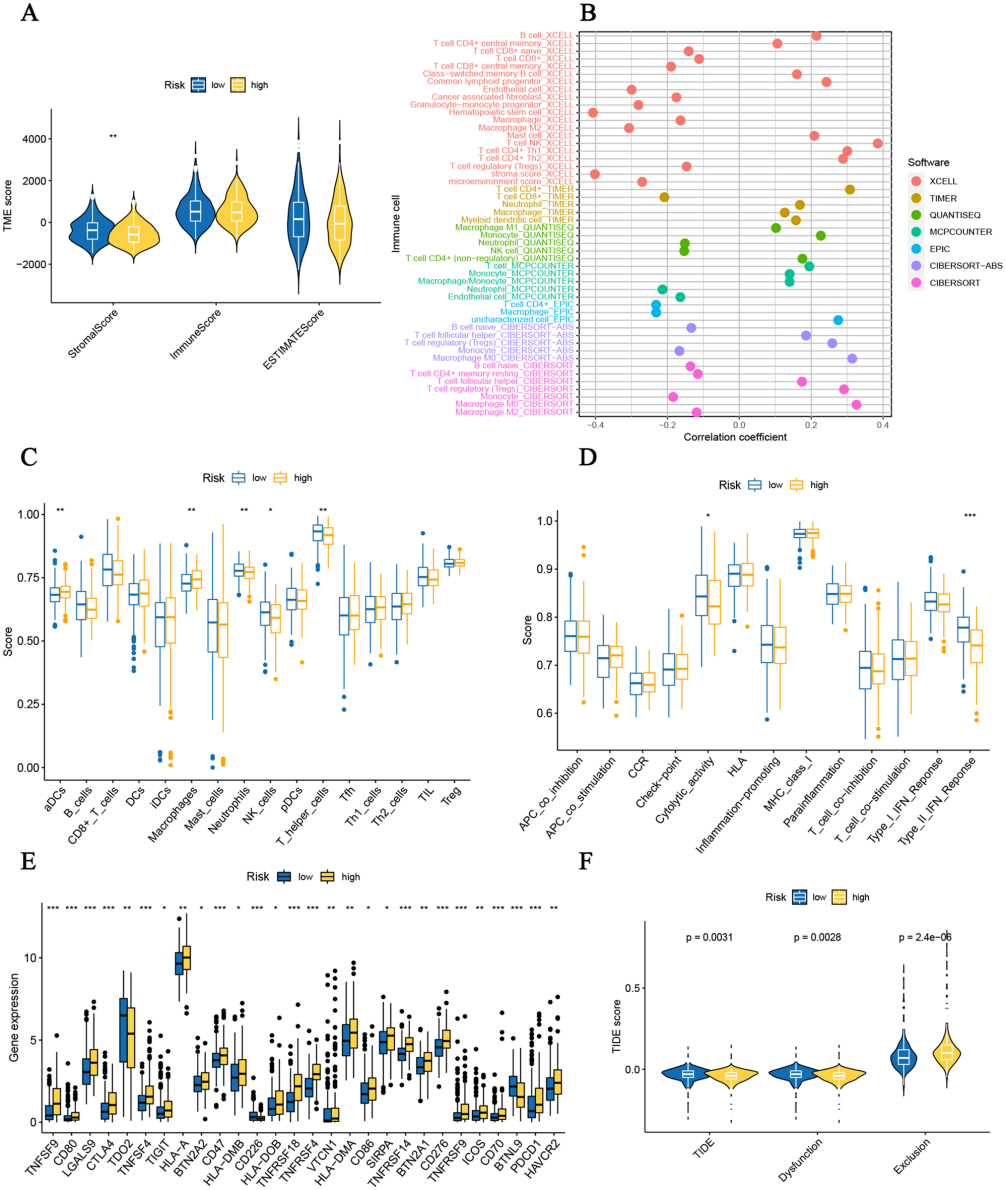
Immune-related analyses of cuproptosis‐related ICGs prognostic signature. (**A**) TME score prediction. (**B**) The immune cell infiltration by multiple methods. The correlation between the predictive signature and (**C**) 16 immune cells and (**D**) 13 immune-related functions. (**E**) The relationship between prognostic signature and immune checkpoints. (**F**)TIDE score prediction. *p < 0.05; **p < 0.01; ***p < 0.001.

We next employed the *ESTIMATE* method to study the correlation with the immune microenvironment. As illustrated in Fig. 7A, the stromal score is relatively lower in high-risk populations (*p* < 0.01), suggesting low tumor purity in high-risk populations. In addition, immune infiltration scores quantified by multiple algorithms reveal that this 5-CRICG signature was strongly associated with immune cell infiltration (Fig. 7B). Next, we adopted the ssGSEA method to investigate the immune-related functions of this prognostic signature. Compared with the low-risk populations, the high-risk populations had a relatively increased degree of aDCs and macrophage cell infiltration and a moderately decreased degree of Neutrophils, T helper cells, and natural killer (NK) cells infiltration (Fig. 7C). In addition, the high-risk populations had rather attenuated activity in several immune functions in terms of type II IFN response and cytolytic activity (Fig. 7D). Besides, the other immune checkpoint gene expression levels among these two populations were compared. As shown in Fig. 7E, 28 immune checkpoint genes (including these 5 ICGs) were differentially expressed. Various immune checkpoint genes as effective immunotherapy targets are highly expressed in high-risk populations, including CTLA4, TIGIT, and CD276 (B7-H3). Notably, the TIDE scores (*p* = 0.0031) and dysfunction scores (*p* = 0.0028) were relatively lower in the high-risk populations, while the exclusion scores (*p* < 0.001) were significantly higher, indicating that the high-risk populations might correlate with better ICB responses (Fig. 7F).

### Drug susceptibility analysis

After that, we screened 10 chemotherapeutic or targeted drugs through drug sensitivity comparison (Fig. S3). The drug sensitivity results showed that the high-risk population was more resistant to Elesclomol (cuproptosis inducer), verifying the consistency of this model signature. In addition, the high-risk populations were more resistant to Camptothecin (DNA topoisomerase I inhibitor), Axitinib (multi-targeted tyrosine kinase inhibitor), Erlotinib (EGFR tyrosine kinase inhibitor), and Gefitinib (EGFR tyrosine kinase inhibitor); and more sensitive to 5-Fluorouracil (DNA/RNA Synthesis inhibitor), Doxorubicin (DNA Topoisomerase inhibitor), Mitomycin C (DNA cross-linking agent), Paclitaxel (microtubule-associated inhibitor), and Sunitinib (multi-targeted receptor tyrosine kinase inhibitor).

### Somatic mutation and TMB analysis

**Figure 8:**
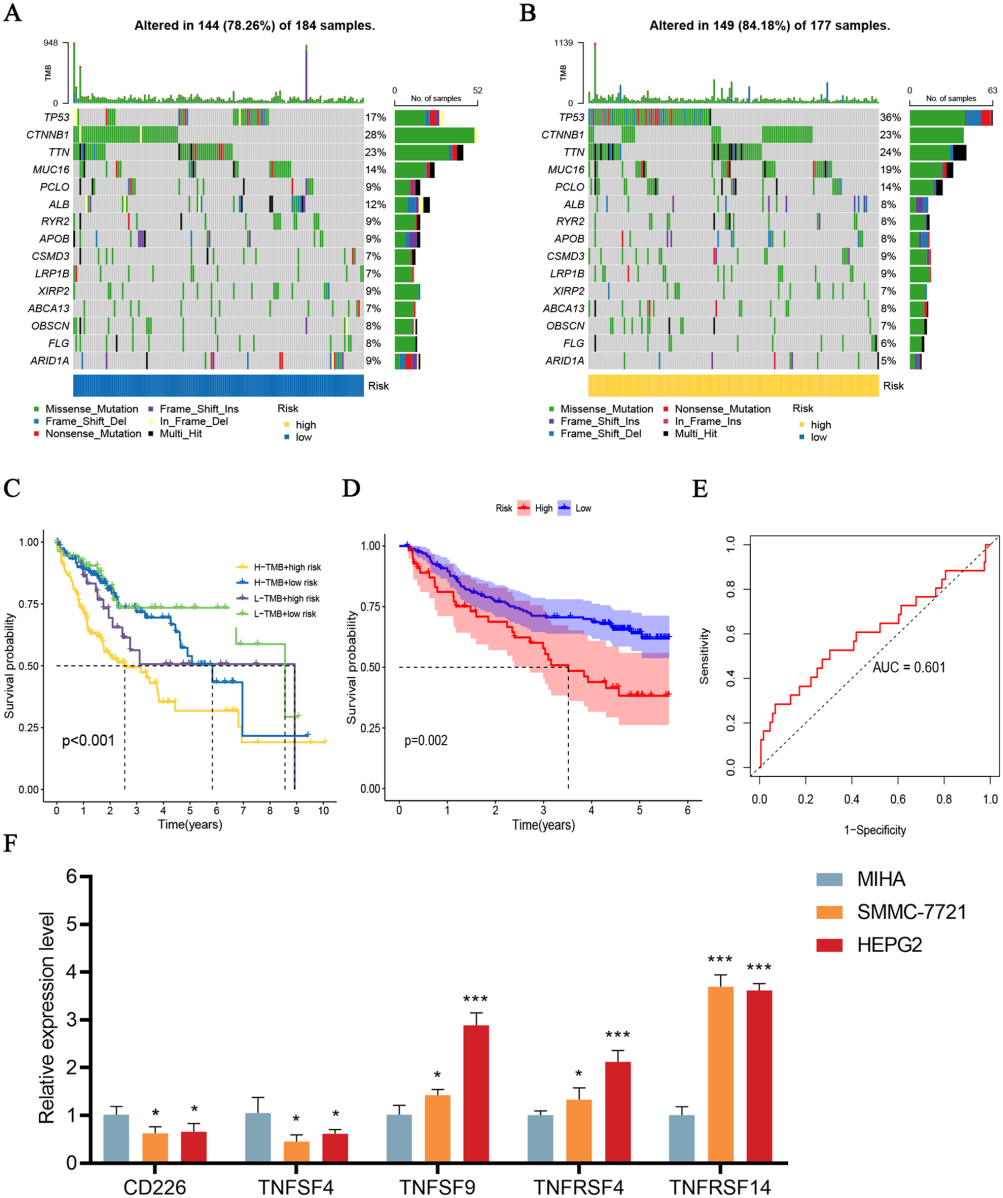
Somatic mutation analysis and external validation of cuproptosis‐related ICGs model. (**A**, **B**) The waterfall plot showing the difference in somatic mutation features between the low- and high-risk populations. (**C**) Kaplan–Meier survival curves of the prognostic signature combined with TMB. (**D**) OS in the GSE14520 dataset. (**E**) The ROC curves for 1-year OS in the GSE14520 dataset. (**F**) Expression levels of TNFRSF4, TNFRSF14, TNFSF4, TNFSF9, and CD226 in HCC cell lines. *p < 0.05; ***p < 0.001.

Subsequently, we evaluated the somatic mutation status among the high- and low-risk populations. The frequency top 10 mutated genes were TP53, CTNNB1, TTN, MUC16, PCLO, ALB, RYR2, APOB, CSMD3, and LRP1B (Fig. 8A,B). Among them, the frequencies of gene mutations in TP53, MUC16, PCLO, CSMD3, and LRP1B were increased in the high-risk populations. Besides, a significant difference was shown in the survival analysis of the risk groups combined with TMB (*p* < 0.001, Fig. 8C). The high-risk populations combined with high TMB scores had the worst OS.

### External validation of prognostic signature

We externally validated this prognostic signature with the GSE14520 dataset. K–M survival curves for OS indicate that high-risk populations strongly correlate with unfavorable outcomes (*p* = 0.002, Fig. 8D). The AUC of 1-year OS was 0.601 (Fig. 8E). The PFS analysis yielded identical results (*p* = 0.005, Fig. S2B). Thus, we further evaluated the expression levels of TNFRSF4, TNFRSF14, TNFSF4, TNFSF9, and CD226 in the HCC cell lines. We observed significant downregulation of CD226 and TNFRSF4 in HCC cell lines, as well as prominent upregulation of TNFRSF14, TNFSF4, and TNFSF9 (Fig. 8F).

## Discussion

Cu is an important metallic trace element in the human body and is engaged in various biochemical functions such as mitochondrial respiration, iron absorption, autophagy, and antioxidant defense^33^. The liver is the main organ responsible for removing Cu from the human body^34,35^. In hepatocytes, the Cu excretion is primarily mediated by the copper exporter ATP7A/B^36–39^. The disorders of Cu metabolism are closely related to liver diseases, like Wilson’s disease and even hepatocarcinoma. Elevated levels of Cu are closely correlated with various malignant cancers. The Cu imbalance plays a prominent role in cancer development^40^. In particular, the Cu level may increase the incidence of hepatocarcinoma in Wilson’s disease patients^41^.

A growing number of studies have explored predictive models for ICGs as potential predictors in multiple cancers. Li et al.^42^ investigated a signature of 7 ICGs to predict survival outcomes and therapeutic responses for endometrial carcinoma patients. Liu et al.^43^ identified a signature of 3 ICGs to predict prognosis and immune status in triple-negative breast cancer. The latest research study by Tsvetkov et al. revealed a novel form of Cu-dependent programmed death named cuproptosis. Elevated Cu levels in tumor cells contribute to immune escape by enhancing PD-L1 expression. Thus, studying the connection of cuproptosis with other ICGs is of great interest. Here, we developed a novel prognostic CRICG model, providing a new theoretical foundation for CRICG targets as the future hepatocarcinoma therapy.

Firstly, we focused on 13 CRICGs based on Tsvetkov et al.^16^ research. These genes included 7 cuproptosis resistant genes identified by whole-genome CRISPR-Cas9 positive selection screening. In addition, 3 Cu exporters or importers (ATP7A, ATP7B, and SLC31A1) could regulate cuproptosis sensitivity by affecting intracellular Cu levels^16^. The other 3 critical enzymes in the TCA cycle (DBT, GCSH, and DLST) were also included. Alterations in the expression and catalytic activity of essential enzymes of the TCA cycle are associated with the development of HCC^44,45^.

According to the LASSO-COX regression analyses, we developed a prognostic signature based on 5-CRICGs (TNFRSF4, TNFRSF14, TNFSF4, TNFSF9, and CD226). This prognostic signature was strongly related to the TNM stage, T stage, M stage, and grade of HCC. GO and KEGG analyses showed that the DEGs between these two populations enriched in several biological functions like cell division, DNA replication, and drug response. GSEA results reveal significant differences between these two populations enriched in various tumor-associated signaling pathways, including cell cycle, DNA replication, p53 signaling pathway, and DNA repair pathways. The above results suggest that this signature is remarkably correlated with the tumorigenesis and progression of HCC.

Again, we investigated the potential connection between the prognosis model and the immune landscape. According to the multiple immune infiltration quantification algorithms, this 5-CRICGs signature was strongly related to the immune cell infiltration. Analyzed by the ssGSEA method, this prognosis signature was attenuated in several immune cell infiltration (including neutrophils, NK cells, and T helper cells) and multiple immune functions in terms of cytolytic activity and type II IFN response. It has been evidenced that type II IFN (also known as IFNγ) could suppress tumor growth by affecting immune cells and tumor cells^46^. The loss of IFNγ is also pinpointed as related to ICB response^47^. These results suggest that the high-risk populations might be in an immunosuppressive state by modulating the IFN response. High-risk populations had prominently lower TIDE scores, indicating that the high-risk populations might correlate with preferable ICB responses. In addition, the drug sensitivity prediction of this CRICG prognostic model in response to common chemotherapeutic or targeted drugs can provide the possibility of personalized treatment for HCC patients.

Finally, the GSE14520 dataset externally validated the prognostic value of this CRICG signature. Furthermore, the expression levels of these 5 CRICGs in the HCC cell lines were detected. These 5 CRICGs were characterized as co-stimulatory molecules in the immune response^48^. CD226 and TNFRSF4 were expressed relatively lower in HCC cell lines, while TNFRSF14, TNFSF4, and TNFSF9 were the opposite trend. Except for TNFSF4, the HCC cell lines expression differences of CD226, TNFSF9, TNFRSF4, and TNFRSF14 correspond to Fig. 2B above. CD226, famous for competing with TIGIT, is an important co-stimulator that regulates T cell and NK cell function^49^. It was demonstrated that CD226 could induce IFNγ generation^50^ and promote HCC cell lysis^51,52^. Recent studies have found that CD226 expression is essential for PD-(L)1 or TIGIT immunotherapy response^53,54^. TNFSF9, also known as CD137L or 4-1BBL, was reported to be upregulated in primary biliary cirrhosis^55^. High TNFRSF14 expression was considered associated with colorectal liver metastasis^56^. Recently, TNFRSF4 (OX40) has emerged as a potential target for cancer immunotherapy^57^. TNFRSF4 binds its ligand TNFSF4 (OX40L) and transmits co-stimulatory signals^58^. The up-regulation of TNFRSF4^59–61^ and down-regulation of TNFSF4^61^ in our HCC cell lines experiment were consistent with the previous studies.

Here, we constructed and validated a prognostic risk model based on 5 CRICGs. This risk model is feasible and effective. However, our research has some limitations. First, the detailed information on treatment options and therapeutic responses of HCC patients in the TCGA database is unavailable. In addition, we only applied the cell lines for expression level validation due to the acquisitiveness constraints of tissue samples and commercial microarrays. Finally, the biological functions and potential mechanisms of these ICGs in hepatocellular carcinoma have not been explored in depth. Furtherly, we will continue to collect clinical tissue samples and design more in vitro experiments to investigate the function of these prognostic ICGs.

## Conclusions

Our prognostic signature based on 5 CRICGs could validly forecast the outcomes and immune response of HCC patients, and is promising as biomarkers to be developed for anticancer therapy.

## Supplementary Information

Supplementary Figures.

## Data Availability

The datasets used and/or analyzed during the current study are available from the corresponding author on reasonable request.

## References

[1] Siegel RL, Miller KD, Fuchs HE, Jemal A (2021). Cancer statistics, 2021. CA Cancer J. Clin..

[2] He Y (2021). Biomarkers and future perspectives for hepatocellular carcinoma immunotherapy. Front. Oncol..

[3] Pinter M, Jain RK, Duda DG (2021). The current landscape of immune checkpoint blockade in hepatocellular carcinoma: A review. JAMA Oncol..

[4] Leone P (2021). The evolving role of immune checkpoint inhibitors in hepatocellular carcinoma treatment. Vaccines.

[5] Członkowska A (2018). Wilson disease. Nat. Rev. Dis. Prim..

[6] Tsvetkov P (2019). Mitochondrial metabolism promotes adaptation to proteotoxic stress. Nat. Chem. Biol..

[7] Ge EJ (2022). Connecting copper and cancer: From transition metal signalling to metalloplasia. Nat. Rev. Cancer.

[8] Baltaci AK, Dundar TK, Aksoy F, Mogulkoc R (2017). Changes in the serum levels of trace elements before and after the operation in thyroid cancer patients. Biol. Trace Elem. Res..

[9] Stepien M (2017). Pre-diagnostic copper and zinc biomarkers and colorectal cancer risk in the European prospective investigation into cancer and nutrition cohort. Carcinogenesis.

[10] Zhang X, Yang Q (2018). Association between serum copper levels and lung cancer risk: A meta-analysis. J. Int. Med. Res..

[11] Aubert L (2020). Copper bioavailability is a KRAS-specific vulnerability in colorectal cancer. Nat. Commun..

[12] Saleh SAK, Adly HM, Abdelkhaliq AA, Nassir AM (2020). Serum levels of selenium, zinc, copper, manganese, and iron in prostate cancer patients. Curr. Urol..

[13] Michniewicz F (2021). Copper: An intracellular achilles' heel allowing the targeting of epigenetics, kinase pathways, and cell metabolism in cancer therapeutics. ChemMedChem.

[14] Babak MV, Ahn D (2021). Modulation of intracellular copper levels as the mechanism of action of anticancer copper complexes: Clinical relevance. Biomedicines.

[15] Wang Y, Zhang L, Zhou F (2022). Cuproptosis: A new form of programmed cell death. Cell. Mol. Immunol..

[16] Tsvetkov P (2022). Copper induces cell death by targeting lipoylated TCA cycle proteins. Science.

[17] Tang D, Chen X, Kroemer G (2022). Cuproptosis: A copper-triggered modality of mitochondrial cell death. Cell Res..

[18] O'Dell BL (1993). Interleukin-2 production is altered by copper deficiency. Nutr. Rev..

[19] Prohaska JR, Lukasewycz OA (1981). Copper deficiency suppresses the immune response of mice. Science.

[20] Jones DG (1984). Effects of dietary copper depletion on acute and delayed inflammatory responses in mice. Res. Vet. Sci..

[21] Voli F (2020). Intratumoral copper modulates PD-L1 expression and influences tumor immune evasion. Can. Res..

[22] Hu FF, Liu CJ, Liu LL, Zhang Q, Guo AY (2021). Expression profile of immune checkpoint genes and their roles in predicting immunotherapy response. Brief. Bioinform..

[23] Friedman J, Hastie T, Tibshirani R (2010). Regularization paths for generalized linear models via coordinate descent. J. Stat. Softw..

[24] Kamarudin AN, Cox T, Kolamunnage-Dona R (2017). Time-dependent ROC curve analysis in medical research: Current methods and applications. BMC Med. Res. Methodol..

[25] Kanehisa M, Furumichi M, Sato Y, Ishiguro-Watanabe M, Tanabe M (2021). KEGG: Integrating viruses and cellular organisms. Nucleic Acids Res..

[26] Yoshihara K (2013). Inferring tumour purity and stromal and immune cell admixture from expression data. Nat. Commun..

[27] Aran D, Sirota M, Butte AJ (2015). Systematic pan-cancer analysis of tumour purity. Nat. Commun..

[28] Sturm G (2019). Comprehensive evaluation of transcriptome-based cell-type quantification methods for immuno-oncology. Bioinformatics.

[29] Rooney MS, Shukla SA, Wu CJ, Getz G, Hacohen N (2015). Molecular and genetic properties of tumors associated with local immune cytolytic activity. Cell.

[30] Jiang P (2018). Signatures of T cell dysfunction and exclusion predict cancer immunotherapy response. Nat. Med..

[31] Mayakonda A, Lin DC, Assenov Y, Plass C, Koeffler HP (2018). Maftools: Efficient and comprehensive analysis of somatic variants in cancer. Genome Res..

[32] Jeppesen DK, Bohr VA, Stevnsner T (2011). DNA repair deficiency in neurodegeneration. Prog. Neurobiol..

[33] Ruiz LM, Libedinsky A, Elorza AA (2021). Role of copper on mitochondrial function and metabolism. Front. Mol. Biosci..

[34] Wijmenga C, Klomp LW (2004). Molecular regulation of copper excretion in the liver. Proc. Nutr. Soc..

[35] Chen J (2020). The molecular mechanisms of copper metabolism and its roles in human diseases. Pflugers Arch..

[36] Linder MC (2020). Copper homeostasis in mammals, with emphasis on secretion and excretion: A review. Int. J. Mol. Sci..

[37] La Fontaine S, Mercer JF (2007). Trafficking of the copper-ATPases, ATP7A and ATP7B: Role in copper homeostasis. Arch. Biochem. Biophys..

[38] Roelofsen H (2000). Copper-induced apical trafficking of ATP7B in polarized hepatoma cells provides a mechanism for biliary copper excretion. Gastroenterology.

[39] Polishchuk EV (2014). Wilson disease protein ATP7B utilizes lysosomal exocytosis to maintain copper homeostasis. Dev. Cell.

[40] Shanbhag VC (2021). Copper metabolism as a unique vulnerability in cancer. Biochim. et Biophys. Acta Mol. Cell Res..

[41] Vanderwerf SM, Cooper MJ, Stetsenko IV, Lutsenko S (2001). Copper specifically regulates intracellular phosphorylation of the Wilson's disease protein, a human copper-transporting ATPase. J. Biol. Chem..

[42] Li S (2021). Identification of an immune checkpoint gene signature that accurately predicts prognosis and immunotherapy response in endometrial carcinoma. Aging.

[43] Liu J (2022). A novel immune checkpoint-related gene signature for predicting overall survival and immune status in triple-negative breast cancer. Transl. Cancer Res..

[44] Todisco S, Convertini P, Iacobazzi V, Infantino V (2019). TCA Cycle rewiring as emerging metabolic signature of hepatocellular carcinoma. Cancers.

[45] Wan S (2015). Polymorphisms in genes of tricarboxylic Acid cycle key enzymes are associated with early recurrence of hepatocellular carcinoma. PLoS ONE.

[46] Fenton SE, Saleiro D, Platanias LC (2021). Type I and II interferons in the anti-tumor immune response. Cancers.

[47] Gao J (2016). Loss of IFN-γ pathway genes in tumor cells as a mechanism of resistance to anti-CTLA-4 therapy. Cell.

[48] Yu X, Zheng Y, Mao R, Su Z, Zhang J (2019). BTLA/HVEM signaling: Milestones in research and role in chronic hepatitis B virus infection. Front. Immunol..

[49] Lozano E, Dominguez-Villar M, Kuchroo V, Hafler DA (2012). The TIGIT/CD226 axis regulates human T cell function. J. Immunol..

[50] Shibuya K (2003). CD226 (DNAM-1) is involved in lymphocyte function-associated antigen 1 costimulatory signal for naive T cell differentiation and proliferation. J. Exp. Med..

[51] Conner M, Hance KW, Yadavilli S, Smothers J, Waight JD (2022). Emergence of the CD226 Axis in cancer immunotherapy. Front. Immunol..

[52] Toutirais O (2009). DNAX accessory molecule-1 (CD226) promotes human hepatocellular carcinoma cell lysis by Vgamma9Vdelta2 T cells. Eur. J. Immunol..

[53] Banta KL (2022). Mechanistic convergence of the TIGIT and PD-1 inhibitory pathways necessitates co-blockade to optimize anti-tumor CD8(+) T cell responses. Immunity.

[54] Weulersse M (2020). Eomes-dependent loss of the Co-activating receptor CD226 restrains CD8(+) T cell anti-tumor functions and limits the efficacy of cancer immunotherapy. Immunity.

[55] Xia R (2010). TNFSF9 expression in primary biliary cirrhosis and its clinical significance. Cytokine.

[56] Sasaki Y (2019). Significance of herpesvirus entry mediator expression in human colorectal liver metastasis. Ann. Surg. Oncol..

[57] Fu Y, Lin Q, Zhang Z, Zhang L (2020). Therapeutic strategies for the costimulatory molecule OX40 in T-cell-mediated immunity. Acta Pharmaceutica Sin. B.

[58] Webb GJ, Hirschfield GM, Lane PJ (2016). OX40, OX40L and autoimmunity: A comprehensive review. Clin. Rev. Allergy Immunol..

[59] Piconese S (2014). Human OX40 tunes the function of regulatory T cells in tumor and nontumor areas of hepatitis C virus-infected liver tissue. Hepatology.

[60] Xie K (2018). OX40 expression in hepatocellular carcinoma is associated with a distinct immune microenvironment, specific mutation signature, and poor prognosis. Oncoimmunology.

[61] Du P, Wang Z, Geng J, Wang Y (2021). Expression and clinical significance of OX40 and OX40L mRNA in hepatocellular carcinoma. Bull. Exp. Biol. Med..

