# Supplementary material for: A novel cuproptosis-related immune checkpoint gene signature identification and experimental validation in hepatocellular carcinoma
Source: Sci Rep. 2022 Nov 2;12:18514. doi: 10.1038/s41598-022-22962-y (PMC9630496; doi:10.1038/s41598-022-22962-y)
Supplement: Supplementary file 1 — Supplementary Figures. [file 41598_2022_22962_MOESM1_ESM.pdf]

A Novel Cuproptosis-Related Immune Checkpoint Gene Signature Identification and Experimental Validation in Hepatocellular Carcinoma

Yusai Xie, Wei Zhang, Jia Sun, Lingyan Sun, Fanjie Meng, and Huiying Yu

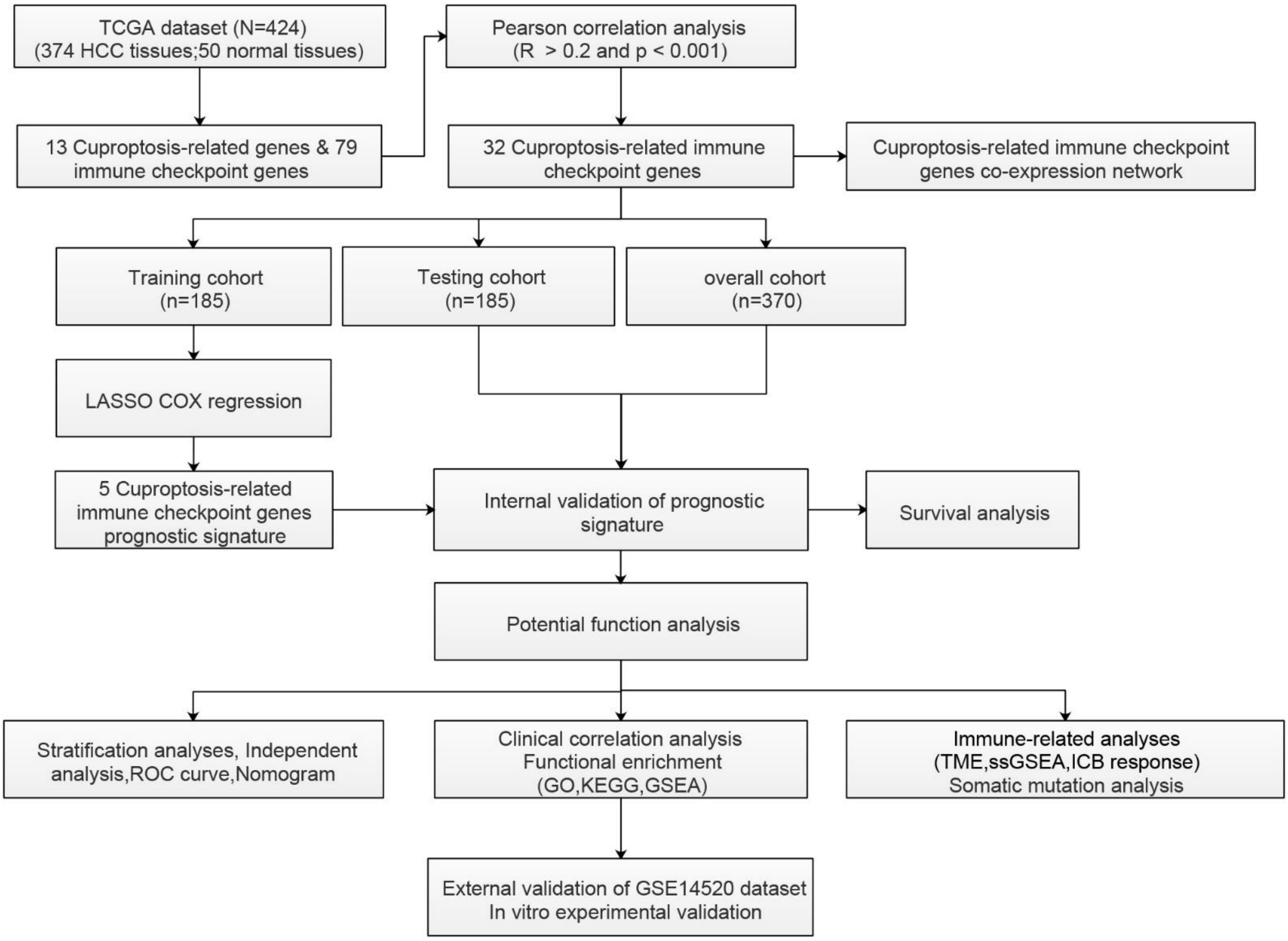

Figure S1. Flowchart of the study.

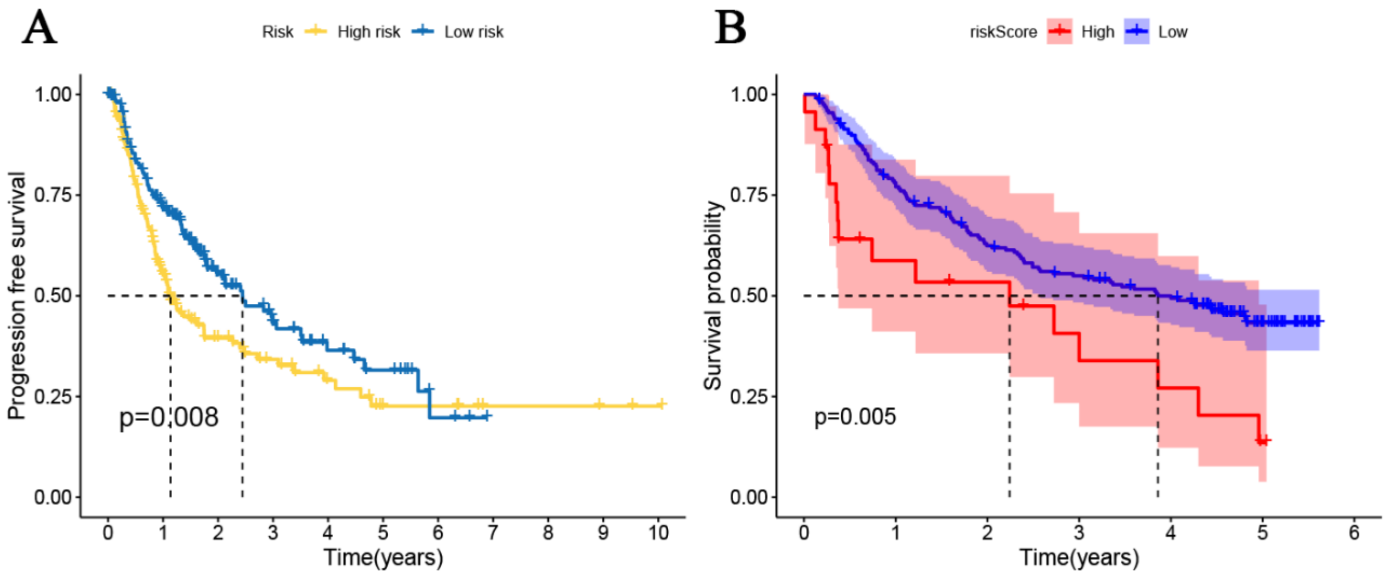

Figure S2. PFS value of (A) TCGA-HCC dataset and (B) GSE14520 dataset.

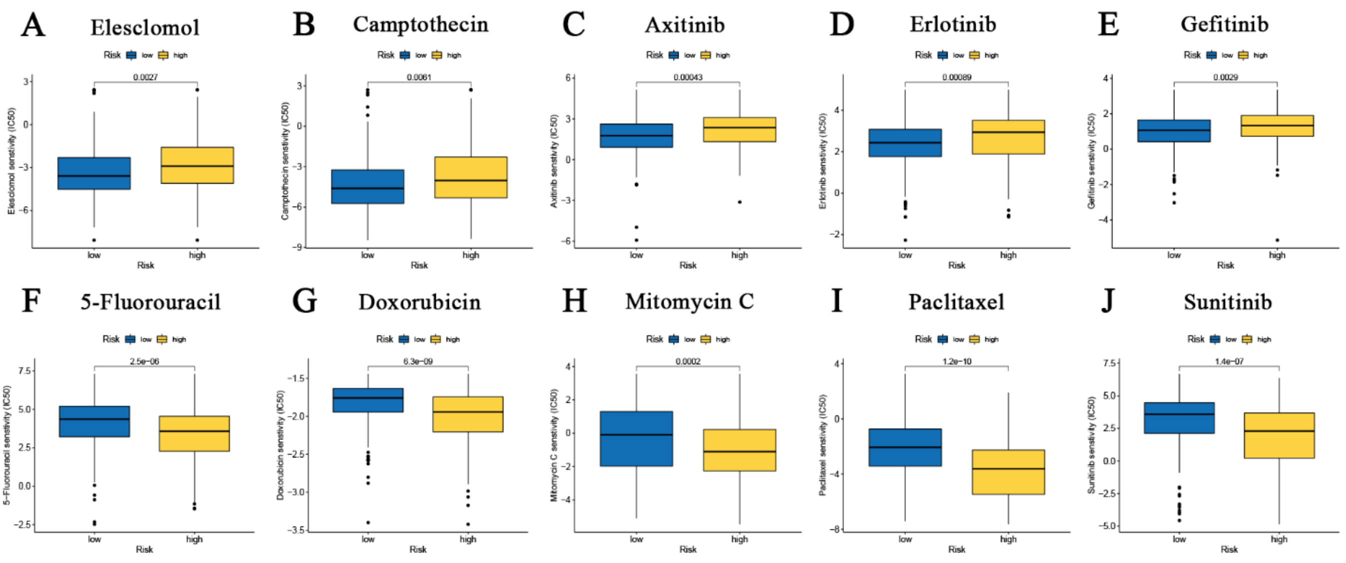

Figure S3. The IC50 difference of chemotherapeutic or targeted drugs in low- and high-risk populations.
